# Can Humic Water Discharge Counteract Eutrophication in Coastal Waters?

**DOI:** 10.1371/journal.pone.0061293

**Published:** 2013-04-18

**Authors:** Agneta Andersson, Iveta Jurgensone, Owen F. Rowe, Paolo Simonelli, Anders Bignert, Erik Lundberg, Jan Karlsson

**Affiliations:** 1 Department of Ecology and Environmental Science, Umeå University, Umeå, Sweden; 2 Latvian Institute of Aquatic Ecology, Marine Monitoring Centre, Riga, Latvia; 3 Department of Biology, University of Bergen, Bergen, Norway; 4 Swedish Museum of Natural History, Stockholm, Sweden; 5 Umeå Marine Sciences Centre, Umeå University, Hörnefors, Sweden; Institute of Marine Research, Norway

## Abstract

A common and established view is that increased inputs of nutrients to the sea, for example via river flooding, will cause eutrophication and phytoplankton blooms in coastal areas. We here show that this concept may be questioned in certain scenarios. Climate change has been predicted to cause increased inflow of freshwater to coastal areas in northern Europe. River waters in these areas are often brown from the presence of high concentrations of allochthonous dissolved organic carbon (humic carbon), in addition to nitrogen and phosphorus. In this study we investigated whether increased inputs of humic carbon can change the structure and production of the pelagic food web in the recipient seawater. In a mesocosm experiment unfiltered seawater from the northern Baltic Sea was fertilized with inorganic nutrients and humic carbon (CNP), and only with inorganic nutrients (NP). The system responded differently to the humic carbon addition. In NP treatments bacterial, phytoplankton and zooplankton production increased and the systems turned net autotrophic, whereas the CNP-treatment only bacterial and zooplankton production increased driving the system to net heterotrophy. The size-structure of the food web showed large variations in the different treatments. In the enriched NP treatments the phytoplankton community was dominated by filamentous >20 µm algae, while in the CNP treatments the phytoplankton was dominated by picocyanobacteria <5 µm. Our results suggest that climate change scenarios, resulting in increased humic-rich river inflow, may counteract eutrophication in coastal waters, leading to a promotion of the microbial food web and other heterotrophic organisms, driving the recipient coastal waters to net-heterotrophy.

## Introduction

The efficiency of pelagic food webs determines the amount of energy produced at the highest trophic levels and is highly dependent on the pathway through which energy and matter flows from basal trophic levels (phytoplankton and bacteria) to the top predators (e.g. fish) [1]. Phytoplankton are autotrophs which use inorganic carbon as their carbon source, while heterotrophic bacteria, utilize organic carbon from autochthonous or allochthonous sources. Both are osmotrophic organisms, competing for inorganic nutrients via diffusion into their cells. The relative importance of these functional groups differs between oligotrophic and eutrophic waters [2], and between autochthonous aquatic systems and those influenced by terrestrial run-off [3]. In general, the importance of bacteria is relatively high in low productive areas influenced by allochthonous organic material, while in more productive and eutrophic areas phytoplankton dominate. Net-heterotrophy has been found to be more pronounced in humic-rich than in clear water systems [4], probably due to the dominance of bacteria which often contribute to the largest fraction of the respiration in pelagic systems [5].

The nutrient regime influences the size spectrum of the basal producers as well as the channeling of carbon in the food web [6]. Due to the relatively large surface to volume ratio of small cells, these have a high affinity for nutrients. At low nutrient concentrations small phytoplankton therefore dominate. Under nutrient-constrained conditions phytoplankton exudates represent a large proportion of the photosynthetically produced organic carbon, because organic carbon production cannot be built into biomass due to lack of e.g. nitrogen (N) or phosphorous (P). Under such conditions as much as 40–60% of the primary production can be squandered as exudates [2]. Exudates consist of simple sugars that are a prime carbon source for bacteria, which, in nutrient poor waters become highly competitive for N and P. In oligotrophic aquatic systems the bacteria to phytoplankton biomass ratio is therefore high (e.g. [2]). Because large cells have higher uptake capacity than smaller cells, high nutrient concentrations will lead to a dominance of relatively large phytoplankton. Since N and P are available to produce new phytoplankton biomass, a smaller proportion of the primary production is lost via exudation [7]. In nutrient rich waters therefore, the phytoplankton to bacterial biomass ratio is high.

Another important mechanism governing the phytoplankton-bacteria balance is ecosystem CNP stoichiometry [8]. In particular it has been observed that if dissolved organic carbon is provided, a food web may switch from dominance of phytoplankton production to dominance of heterotrophic bacterial production. Climate change has been predicted to cause increased precipitation in northern Europe [9], with the consequence of increasing dissolved allochthonous organic carbon inputs into high latitude seas [10]. It is likely that increased runoff of colored dissolved organic matter from the terrestrial system may have especially large effects on ecological function of semi-enclosed marine systems, like the inner Baltic Sea and fjords. High allochthonous organic carbon inputs may have negative effects on phytoplankton growth via two different mechanisms: 1) light climate deterioration, since inflowing organic matter is often brown, i.e. consisting of humic substances; and 2) availability of an external and potentially bioavailable carbon source, which may un-couple bacterioplankton from their dependence on phytoplankton production, allowing bacteria to become dominant producers at the basal trophic level. Since the food web is size-structured (e.g. [1]), those containing smaller cells at the base, e.g. bacteria and small phytoplankton, have more trophic levels compared to food webs with basal levels dominated by larger phytoplankton cells. At each trophic level 70–90% of the consumed carbon is lost due to respiration and sloppy feeding [11]. Therefore more losses may occur in food webs in low productive than in more productive systems. The food web efficiency, defined as the productivity at higher trophic levels normalized to the productivity at the basal level, would thus be higher in more productive than in unproductive areas. On the other hand eutrophication in fresh or brackish waters often leads to a dominance of filamentous cyanobacteria [12], which are toxic, less edible and of poorer nutrient quality for zooplankton (e.g. [13]). Under such conditions food web efficiency may be low. This suggests that food web efficiency shows a hump-shaped relation along nutrient gradients. However, darkening of the seawater may also have direct effects on higher trophic levels, for example induce a shift from visual to non-visual predators that could cause declined fish yield [14], [15].

To test the effect of allochthonous matter on food web production and efficiency, an experiment was performed with two types of enrichments gradients; one with NP (nitrogen and phosphorus) and one with CNP (carbon, nitrogen and phosphorus). Seawater from the northern Baltic Sea was used and N and P were added as inorganic nutrients and C was added in the form of humus. We hypothesized that increasing NP load would increase production at all trophic levels, i.e. cause eutrophication in the recipient seawater; while increasing load of CNP would mainly increase bacterial production and cause net-heterotrophy. Furthermore, we hypothesized that food web efficiency would show a hump-shaped relation to NP addition, due to variations in phytoplankton cell sizes and their edibility. At low NP concentration the phytoplankton would be small and the number of tropic levels numerous, whereas at higher NP concentrations the phytoplankton would be larger and the number of trophic levels fewer, however at very high NP concentrations the phytoplankton would be dominated by relatively inedible filamentous forms leading to low food web efficiency. In the CNP enrichments the food web efficiency would be consistently low due to relatively many trophic levels. The results of the study are used to discuss potential effects of climate change and anthropogenic nutrient load on food web function and productivity in coastal marine systems.

## Materials and Methods

### Allochthonous Carbon

Before the start of the experiment we searched for a suitable allochthonous carbon source. To select a representative substance, a bacterial growth experiment was performed where four different commercially available carbon sources were compared to allochthonous carbon isolated from river water entering the northern Baltic Sea. Seawater from the northern Baltic Sea (salinity ∼4 and temperature ∼14°C) was filtered through a 0.65 µm polycarbonate filter to isolate the bacterial fraction and 180 ml was put into each of twenty-one Whirl-Pak bags. The water in the bags was enriched with an NP solution corresponding to thirty percent of the winter concentrations in the northern Baltic Sea (1.97 µM nitrate, 0.33 µM ammonium and 0.23 µM phosphate). In accordance with the Redfield ratio, 23 µM of the different carbon substances were added to three of the bags, respectively. The carbon substances tested were glucose, lactose, humic acid-Aldrich (HA), humic acid-Fluka (HF) and natural fulvic acid isolated from the Öre River (FO). The latter was isolated and purified using adsorption on both DEAE-cellulose and XAD-8 resin [16]. The humic substance was isolated as dried powder and the molecular weight was approximately 1500. Three Whirl-Pak bags were established as controls, where carbon was not added. Incubation was carried out for four days and 15 ml samples for bacterial enumeration were taken each day; preserved with 1% glutaraldehyde, filtered to attach bacteria to filters, stained, and analyzed using epifluorescence microscopy (see bacterial counts below). Growth rates were determined for each of the three replicates from log-normal growth curves. Average values and standard deviation were calculated for the six treatments.

Carbon and nitrogen content of FO, HA and HF were measured by analyzing duplicate samples (5–10 mg) in a Carlo Erba model 1108 high temperature combustion elemental analyzer, following standard procedures and a combustion temperature of 1030°C. Acetanilide was utilized for standardization.

The absorption of photosynthetically active radiation (PAR, 400–700 nm) by natural FO and HF were tested using a scanning spectrophotometer (Thermo Fisher, Evolution 600; slit width 1 nm) in seawater adjusted with humic substances, prior to microbial growth incubation.

### Mesocosm Experiment

The experiment was performed during the late autumn period, October-November, at Umeå Marine Sciences Centre in 19 mesocosm tanks (1 m diameter, 0.5 m high, ∼400 litres), using unfiltered seawater from the northern Baltic Sea (salinity 3.7 PSU). Seawater was collected in the beginning of October at a depth of 4 m in the Bothnian Sea (63° 32,086 N, 019° 56,160 E) using a peristaltic pump (Nilo), and the *in situ* temperature was 11°C. Water was stored in four 2.5 m^3^ clean plastic tanks onboard the ship during transportation to the laboratory. The collection process, including transportation to the laboratory, took less than 2 hours. The water was consecutively pumped into the mesocosms, and care was taken to distribute the water equally between tanks, resulting in each tank containing ∼400 litres. This process was completed in less than 3 hours. The experiment was divided into two treatment types, NP and CNP. In the NP treatments (10 tanks) inorganic nitrogen and phosphorus was added in an eight-step gradient, ranging from 0 to 0.269 µmol phosphate l^−1^ d^−1^, from 0 to 0.603 µmol ammonium l^−1^ d^−1^, and from 0 to 3.701 µmol nitrate l^−1^ d^−1^. The treatments were named 0, 5, 10, 15, 20, 25, 30 and 35 NP, indicating the percentage of the winter N and P concentrations that were added daily. The molecular CNP ratio of all NP enrichments was 0∶14∶1. The 10 NP treatment was replicated three times (daily addition of 0.077 µM phosphate, 0.172 µM ammonium, 1.06 µM nitrate). In the CNP treatments (9 tanks), a daily addition of inorganic nitrogen and phosphorus was made as described above along with additions of humic carbon (HF) at 12.75–89.26 µM C d^−1^. The molecular CNP ratio of all CNP enrichments was 300∶14∶1. The CNP enrichment series comprised the levels 5, 10, 15, 20, 25, 30 and 35. The 10 CNP treatment was replicated three times.

All mesocosms were illuminated with metal halogen lamps (Prismalence, colour temperature 4200 k, effect 150W), placed ∼1 m above the tanks, for 12 hours per day. To mix the water, air was gently bubbled at ∼0.4 m depth. The water temperature was ∼15°C and was maintained by controlling the room temperature. The experiment was conducted for five weeks (35 days) and was performed as a semi flow-through system where five litres of water was replaced by GF/F-filtered seawater each day (pumped from 1 km off shore of Umeå Marine Station and of similar physicochemical constitution to the mesocosm water). Water samples were collected at regular intervals in the middle of the tanks, using a Ruttner sampler. Samples were collected on days 6, 14, 21, 28 and 35, henceforth referred to as week 1, 2, 3, 4 and 5, respectively.

#### Light and nutrients

PAR was measured in each tank, week 1–5, using a PAR Licor sensor. PAR measurements were performed at five spatially distributed positions within each mesocosm at 0, 20, 40 and 50 cm depths, obtaining a total of 20 data points per mesocosm. Area and depth weighted average PAR values were then calculated.

Concentrations of dissolved organic carbon (DOC) and particulate organic carbon (POC) were analyzed in week 4 and 5 to confirm that enrichment within the mesocosms had taken place. Approximately 10 ml of water was filtered through a 0.2 µm pore size filter (Gelman Supor®) and acidified with 100 µl of 2 mol l^−1^ HCl before analysis. DOC was measured using a high temperature carbon analyzer (Shimadzu TOC-5000). All materials in contact with the samples, including the filters and filter units, were acid washed with 1.2 mol l^−1^ HCl and rinsed with Milli-Q water prior to use. For analysis of particulate organic carbon (POC), ∼200 ml samples were filtered onto glass fiber filters (Whatman GF/F precombusted for 4 h at 450 C) and analyzed with a Carlo Erba model 1108 high temperature combustion elemental analyzer, using standard procedures and a combustion temperature of 1030 C. Acetanilide was utilized for standardization, and results were corrected for blank filter carbon content.

Total organic carbon (TOC) content was calculated according to:
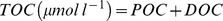



Concentrations of inorganic nutrients (phosphate, nitrate, nitrite, ammonium and silicate) and total nitrogen (Tot N) and phosphorous (Tot P) were analyzed in week 4 and 5, by using a Braan & Luebbe GmbH TRAACS 800 autoanalyzer and standard seawater methods [17]. For analysis of inorganic nutrients, 50 ml of water was filtered through a 0.2 µm cellulose-acetate filter (Gelman Supor®) and kept frozen until analysis. For Tot N and Tot P, unfiltered water was stored frozen until analysis. Dissolved inorganic phosphorous (DIP) was set equal to phosphate concentration and dissolved inorganic nitrogen (DIN) as the sum of nitrate, nitrite and ammonium. Total organic phosphorous (TOP) and total organic nitrogen (TON) were calculated according to:
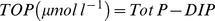


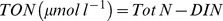



#### Plankton

Mesozooplankton were analyzed from 5.2 l water samples. The zooplankton were collected on a 100 µm mesh and preserved with 0.2% alkaline Lugol’s solution. A Leica stereomicroscope was used to determine the species composition and abundance of mesozooplankton communities. Since the mesozooplankton community was totally dominated by copepods, other groups were ignored in the calculations. Copepods were divided by adults and copepodites in stages CI-III and CIV-V. Lengths were transformed to body mass using the length to weight regressions [18], and assuming a carbon content of 5.2% of the wet weight [19].

Ciliate and phytoplankton samples were preserved with 0.2% (v/v) alkaline Lugol solution and counted under an inverted microscope (Leica DMIL). Ciliate samples (50 ml) were settled in a sedimentation chamber for 24–48 h. Half of the chamber was scanned for ciliates at 200× magnification [20]. For the phytoplankton samples, 10 ml of fixed sample were settled in a sedimentation chamber for 12 h and counted according to the Utermöhl technique at 100× and 400× magnification [21]. Half of the sedimentation chamber was scanned when counting large cells (>10 µm) at 100×magnification. Small cells (<10 µm) were counted at 400× magnification in one diagonal or 20 observed objective fields for more dense samples. Cell sizes were measured using an ocular scale and cell volume calculated according to HELCOM guidelines [22]. Carbon biomasses were calculated according to Menden-Deuer and Lessard [23]. The phytoplankton were divided into three different size groups: <5 µm, 5–20 µm and >20 µm, and their relative contribution to total biomass estimated. The size classes were chosen on the basis of the size of the largest dimension of single cells, filaments or colonies.

Samples for analysis of heterotrophic bacteria, picocyanobacteria and flagellates were preserved with 0.2 µm filtered glutaraldehyde (1% final concentration). For analysis of heterotrophic bacteria, 1–3 ml was filtered onto black 0.2 µm, 25 mm, polycarbonate filters (Poretics®) and stained with acridine orange (0.01% final concentration). Cell abundances and biovolumes were analyzed using an epifluorescence microscope (Nikon TE 300) using blue excitation light connected to an image analysis system [24]. Carbon biomass was calculated according to Norland [25].

For analysis of flagellates and picocyanobacteria 5–10 ml water was filtered onto black 0.6 µm polycarbonate filters (Poretics®), stained with DAPI [26] and counted on a Nikon TE 300 epifluorescence microscope at 1000× magnification. Flagellates were counted in UV light, scanning one diagonal across the filter. Picocyanobacteria were counted by autofluorescence in green excitation light. 100–300 cells per slide were counted.

#### Production

Mesozooplankton net production (*MZp*) was calculated using biomass changes:
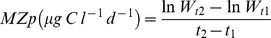
where W_t2_ = Biomass (µg C l^−1^) of copepods at time 2 and W_t1_ = Biomass (µg C l^−1^) of copepods at time 1.

Primary production (Pp) was measured using the ^14^C technique [27]. Triplicate light and one dark sample (each 9 ml) were incubated in acid-washed polycarbonate bottles with 26.8×10^3^ Bq (3.7×10^6^ Bq mmol^−1^) sodium (^14^C) bicarbonate for 4 hours at mean light intensities in the tanks. Samples were then poured into glass scintillation bottles, acidified with 300 µl 6 mol l^−1^ HCl and bubbled with air for 30 min to remove the excess ^14^CO_2_. After adding scintillation cocktail (Optiphase, Hi-Safe 3), samples were analyzed in a Beckman 6500 scintillation counter. Gross (*Pp_g_*) and net (*Pp_n_*) primary production was calculated according to Gargas [27].

Bacterial production (Bp) was measured using the [^3^H]-leucine technique [28] Smith and Azam 1992). Triplicate 1.2 ml samples were incubated for 60 min with 50–100 nM ^3^H-leucine (specific activity 5.66 TBq mmol^−1^). After terminating the incubation by adding 65 µl of 100% TCA, the samples were vortexed, centrifuged (10 min.; 12400 rpm), and the supernatant discarded. The samples were rinsed by the addition of 1.2 ml of 5% TCA, vortexed, centrifuged, and the supernatant discarded. Triplicate controls were pre-killed with 65 µl 100% TCA and treated as described above. Liquid scintillation cocktail (1.2 ml; Opti-Phase 2) was added to the vials and samples were re-suspended by vortexing. Incorporated leucine was measured with a Beckman 6500 scintillation counter and bacterial production was calculated following Simon and Azam [29].

Food web efficiency (*FWE*), defined as mesozooplankton production per basal production (bacterial+primary production) [20], was calculated according to:
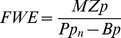



Mesozooplankton net production (*MZp*), net primary production (*Pp_n_*) and net bacterial production (*Bp*) were calculated in µg C l^−1^ d^−1^.

Respiration was measured as oxygen consumption. In each tank one 130 ml flask, filled with mesocosm water, was incubated in the dark for 24 h and oxygen concentrations were measured using the Winkler titration method (Swedish Standard SS-EN 25 813∶1992). Respiration (R) was calculated according to Valiela [30]:
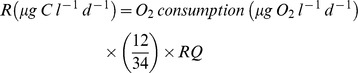



Respiratory quotient (RQ) was set to 1, which has been measured for organisms metabolizing carbohydrates. This value was used for the entire plankton community, although the measurements comprised both autotrophic and heterotrophic organisms.

Net ecosystem production (NEP) was calculated according to:
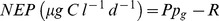



### CNP Concentrations in Rivers and Recipient Estuaries

To relate the nutrient concentrations used in this experiment to that of natural river discharge, we analyzed the concentrations of organic carbon, Tot N and Tot P in three rivers in northern Europe, and their recipient estuaries: the Råne river, entering the Råne estuary (Bothnian Bay, northern Baltic Sea); the Öre river, entering the Öre estuary (Bothnian Sea, northern Baltic Sea) and the Daugava river, entering the Gulf of Riga (southern Baltic Sea). Samples were collected between late May and early June as this period represented free-flowing water conditions. River samples were collected a few kilometers upstream at one to six locations and on one to three different occasions. Estuarine samples were collected at five or six locations on one to three different occasions. Carbon was measured as DOC in the Öre and Råne samples and as TOC in the Daugava river and Gulf of Riga samples. DOC (as above) and TOC are known to give relatively similar values, because the largest pool is DOC (pers. com. E. Lundberg). TOC was measured using a Shimadzu Total Organic Carbon Analyzer TOC – V_CSN._ Total P and N samples were analyzed using standard monitoring methods [22].

### Ethical Consideration

No specific permits were required for the performed field studies in this work. The studied locations are not privately-owned or protected for water sampling. The field studies did not involve endangered or protected species.

### Statistical Analyses

Bacterial growth on different carbon sources was tested using analysis of variance. Differences between groups were tested using a Tpost-hoc test [31].

A paired t-test was used to analyze differences between the NP and the CNP treatments at the two last sampling occasions in the mesocosm experiment, week 4–5. The dependence of bacterial production on primary production was tested for the entire experiment (including both NP and CNP treatments) using regression analysis of a polynomial curve. The dependence of primary production on PAR, Tot N and Tot P was tested for the entire experiment (including both NP and CNP treatments) using multiple regression analysis. The dependence of zooplankton production on primary and bacterial production was tested using multiple regression analysis on average values from the two last sampling occasions. The statistical analyses were carried out with a statistical program, PIA, retrieved from the Arctic Monitoring and Assessment Programme homepage [32].

## Results

### Selection of Model Substance for Allochthonous Carbon

**Figure 1:**
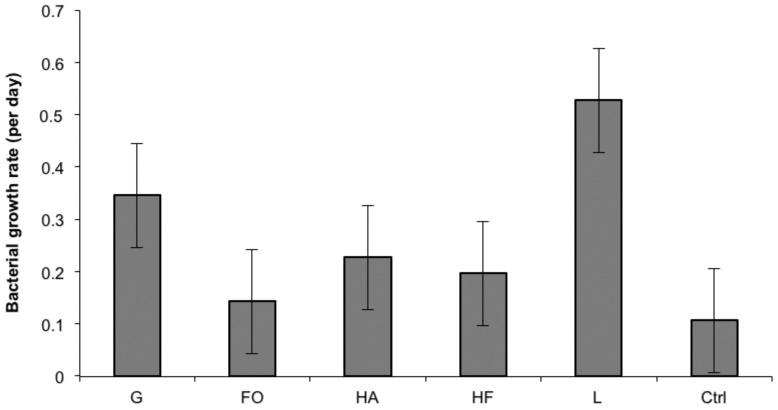
Growth rate of a marine bacterial community on different carbon sources. G = Glucose, FO = Fulvic acid isolated from the Öre river, HA = Humic acid (Aldrich), HF = Humic acid (Fluka), L = Lactose, Ctrl = Control (seawater). Error bars are standard error (n = 3).

The humic acid Fluka (HF) and the natural humic substance FO were found to have similar carbon and nitrogen content; 48% carbon and 0.8% nitrogen, respectively (Table 1). Average bacterial growth rate was ∼30% higher when FO was added to the seawater than in the control (Fig. 1), however, this could not be statistically verified (p>0.05, ANOVA, and T′ post-hoc test, Spjøtvoll and Stoline, 1973). HF and HA addition gave ∼75% (p<0.019) and 100% higher bacterial growth rates than the seawater control, respectively (Fig. 1). The HA assembly was however excluded from the statistical analysis because it had significantly higher variance than the rest of the groups. Additions of the more bio-available carbon sources, lactose and glucose, increased the bacterial growth rates by 200–300% (p<0.001 and p = 0.001, respectively). Absorbance scans showed that HF absorbed 16 times more PAR than FO. HF was chosen as model substance for allochthonous carbon in the mesocosm experiment.

**Table 1:** Carbon and nitrogen content of three humic substances (% of dry weight).

Humic substance	C(% of dw)	N(% of dw)	C:N
Humic acid (Fluka) (HF)	47.6	0.8	60∶1
Humic acid (Aldrich) (HA)	39.2	0.5	72∶1
Fulvic acid (Öre river) (FO)	48.1	0.8	61∶1

Standard deviation of the replicates varied between 1 and 10%.

### Mesocosm Experiment

#### Physical-chemical environment

**Figure 2:**
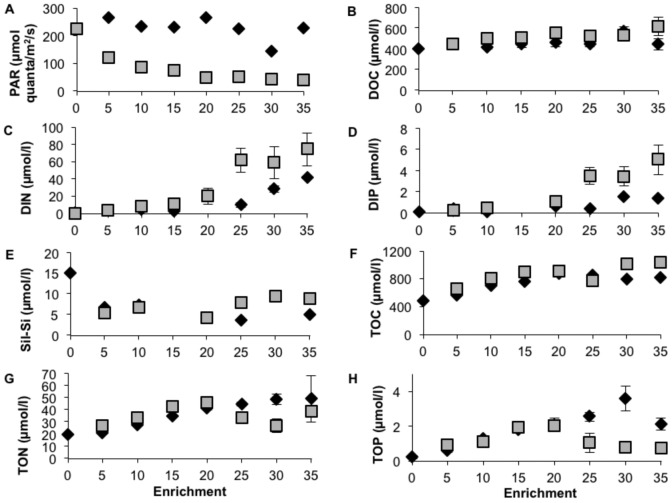
Mean values of PAR light (a), DOC (b), DIN (c), DIP (d) and Sil-Si (e), TOC (f), TON (g) and TOP (h) for NP (⧫) and CNP (▪) treatments sampled during the final two weeks of the mesocosm experiment. Error bars are standard error (n = 2).

The light intensity in the NP mesocosms was on average ∼230 µmol PAR quanta m^−2^ s^−1^. CNP enrichment caused a marked reduction of the PAR, for example in the >20 CNP treatment the average light was reduced by ∼80%, to <50 µmol quanta m^−2^ s^−1^ (Fig. 2a). Paired t-test showed significant differences for the 10–35 NP/CNP levels (n = 16, p<0.001). The average light was relatively stable over time within each treatment (standard deviation 18%).

The concentrations of DOC, TOC, DIN and DIP increased more in the CNP than in the NP enrichments (Fig. 2 b, c, d, f). Paired t-test showed higher concentrations of DOC, DIN and DIP in the CNP treatments at the 10–35 levels (DOC n = 16, p<0.049, DIN n = 10, p<0.008, DIP n = 6, p<0.009), and of TOC for the 5–35 levels (n = 7, p<0.042). The concentrations of TON and TOP, on the other hand, increased more in the NP than in the CNP enrichments (Fig. 2 g, h). Paired t-test showed higher concentrations of TOP in the NP treatments at the 10–35 levels (n = 15, p<0.012). Inorganic silica was highest in the non-enriched treatment (0 NP/CNP). The concentrations tended to decrease more in the NP enrichments, however, statistically significant differences between the NP and CNP treatments could not be found (Fig. 2 e).

#### Plankton biomasses

**Figure 3:**
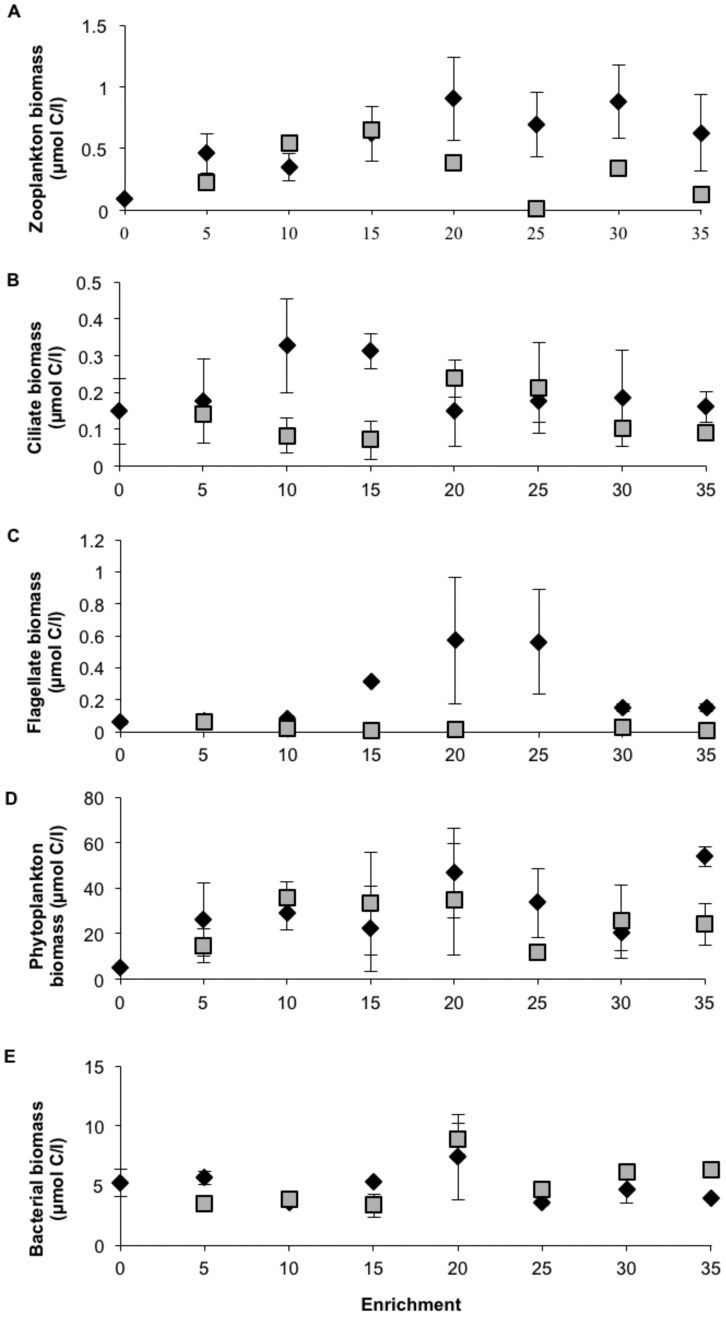
Biomasses of mesozooplankton (a), ciliates (b), flagellates (c), algae (d) and heterotrophic bacteria (e) for NP (⧫) and CNP (▪) treatments sampled during the two last weeks of the mesocosm experiment. Error bars are standard error (n = 2).

Throughout the experiment the mesozooplankton community was dominated by copepods, *Eurytemora affinis* constituting 99% of the total biomass. In almost all of the tanks zooplankton biomass increased during the final two weeks of the experiment (Fig. S1 e and f). During the experiment the abundances of copepods varied from 18 to 107 individuals per liter in the NP additions and from 5 to 88 in the humic treatments. By the end of the experiment (week 4–5), zooplankton biomass showed a clear increase in the NP enrichment gradient, while it only increased up to the mid level (15) in the CNP gradient (Fig. 3a). The NP and CNP treatments differed significantly at the 10–35 level (n = 12, p<0.019).

The ciliate communities were dominated by *Mesodinium pulex, Lohmaniella, Vorticella,* and *Euplotes.* Ciliate biomass peaked during weeks two and three of the experiment in both the NP and CNP treatments (data not shown). In the final two weeks of the experiment (week 4–5), the ciliate biomasses were relatively similar in NP and CNP enrichments with the exception of the 10 and 15 levels (Fig. 3b), where the average values were higher in NP. Overall, the ciliate biomasses did not differ in the NP and CNP treatments at the 10–35 levels (n = 12, p>0.05).

The flagellate community was dominated by nanoplanktonic non-coloured chrysophyceans, cf. *Paraphysomonas* spp. Flagellate abundances showed a general decrease from the first to the second sampling week, and after that their biomass concentration was relatively stable in most tanks (data not shown). During the final two weeks of the experiment (week 4–5), higher values were observed in the >10 NP enrichments than in the corresponding CNP treatments (Fig. 3c, n = 9, p<0.039).

Phytoplankton biomasses increased steadily till the third week after which they levelled off or decreased in the NP and CNP treatments, respectively (Fig. S1 c and d). However, at each time period of the experiment both types of enrichment in general induced higher phytoplankton biomasses than the non-enriched control (Fig. S1 c and d, Fig. 3d).

**Figure 4:**
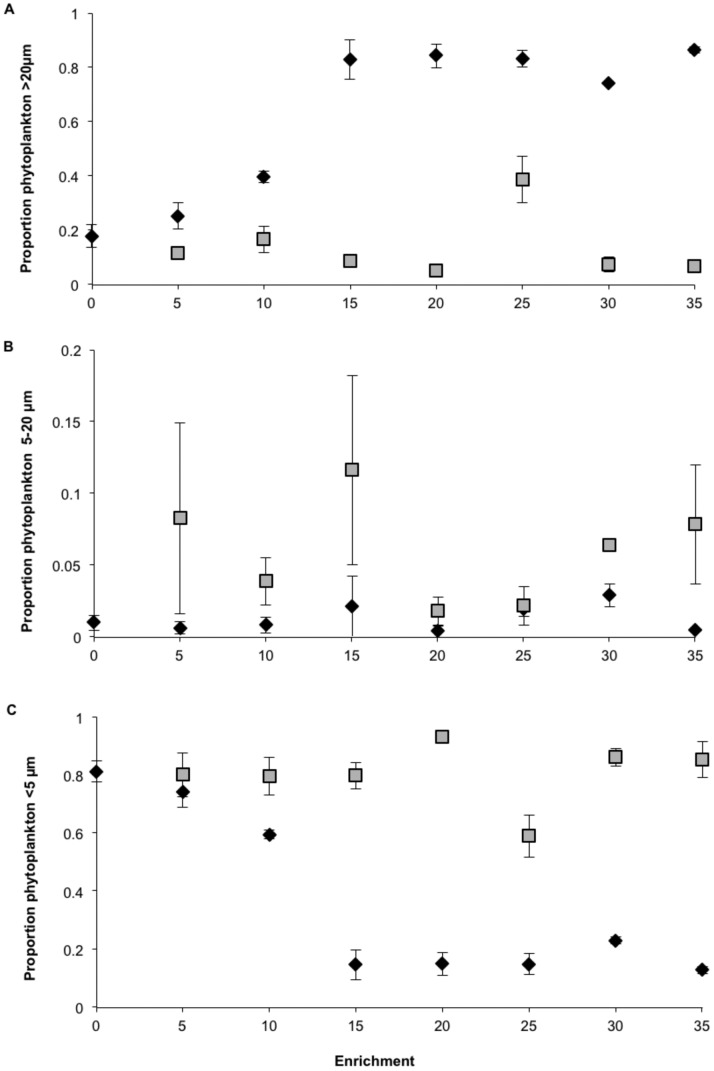
Proportion of phytoplankton biomass >20 µm (a), 5–20 µm (b) and <5 µm (c) for NP (⧫) and CNP (▪) treatments during 2 last weeks of the mesocosm experiment. Error bars are standard error (n = 2).

At the start of the experiment <5 µm phytoplankton dominated the carbon biomass in all tanks, mainly *Synechococcus* spp., constituting ∼60% of the biomass, while 5–20 µm, e.g. *Chrysochromulina* spp. and *Pyramimonas* spp. and >20 µm - *Aphanizomenon flos-aquae* constituted ∼20% each. In the NP enrichments the relative importance of 5–20 µm phytoplankton decreased during the first three weeks and remained close to zero from then on (Fig. S2 a–c). During the final two weeks of the experiment (week 4–5), picocyanobacteria dominated in the 0–10 NP treatments (Fig. 4c, Fig. S2 a), while at higher NP enrichments microplankton >20 µm dominated. These were represented by the colony-forming cyanobacterium *Woronichinia compacta* and the green algae *Ulotrix* spp. and *Oocystis* spp., constituting 70–90% of the biomass (Fig. 4a).

In the CNP enrichments, phytoplankton in the size 5–20 µm, e.g. *Pyramimonas* spp. and *Pseudopediniella* spp. were dominant during the first two or three weeks of the experiment (Fig. S2 d–f), constituting 40–90% of the total phytoplankton biomass across the enrichment gradient. After that this size group decreased to low biomass concentrations. In most of the CNP enrichments large phytoplankton, >20 µm, e.g. *Chaetoceros ceratosporus,* and *Nitzschia intermedia* increased in importance during the first three weeks of the experiment, constituting up to ∼70% of the biomass, there after their biomass diminished. Small phytoplankton <5 µm became dominant in all CNP enrichments over the time course of the experiment, constituting 80–90% of the phytoplankton biomass at the end of the experiment (Fig. 4c, see Fig. S3 for a time-course perspective). Picocyanobacteria were the most important group. Taken together, the proportion of >20 µm, 5–20 µm and <5 µm phytoplankton differed significantly in the NP and CNP treatments at the 5–35 levels (n = 14, p<0.001, n = 13, p<0.006; n = 14, p<0.001, respectively).

Biomasses of heterotrophic bacteria increased over time in most treatments (Fig. S1 a and b). During the final two weeks of the experiment, bacterial biomasses were relatively similar in both enrichment gradients (Fig. 3e). However, in contrast to the other plankton groups average bacterial biomasses tended to be higher in the 20–35 CNP levels compared to the equivalent NP treatments (Fig. 3e).

#### Production rates

**Figure 5:**
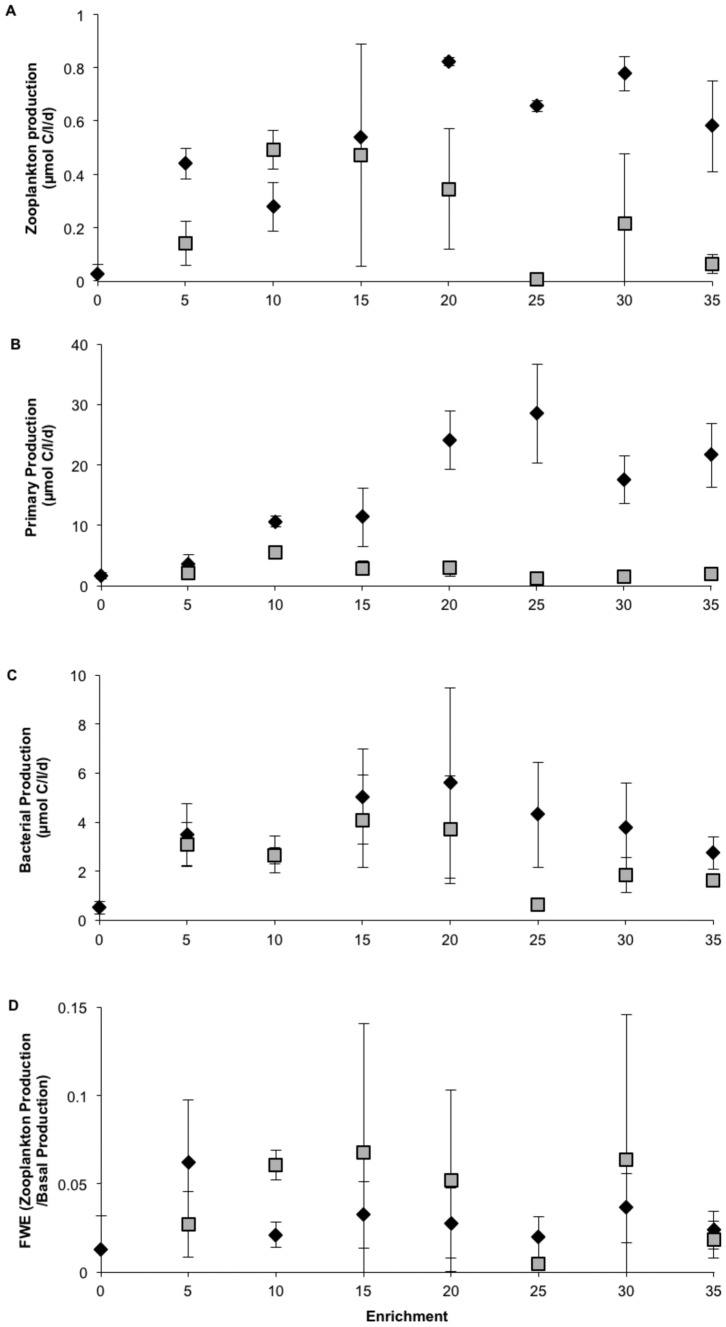
Production rates of mesozooplankton (a), phytoplankton (b) and bacteria (c) for NP (⧫) and CNP (▪) treatments sampled during the two last weeks of the mesocosm experiment. Food web efficiency is presented in panel d. Error bars are standard error (n = 2).

During the two last weeks of the experiment (week 4–5), mesozooplankton production in the NP series showed an increase with enrichment up to the 20 NP level after which it stabilized (Fig. 5a). In the CNP series the highest production was observed at the 10 CNP level (Fig. 5a). Multiple regression analysis showed that as much as 76% of the variation in zooplankton production could be explained by combined variations in primary and bacterial production (n = 19), primary production contributing with 43% (p<0.010) and bacterial production 57% (p<0.001). Zooplankton production was significantly different in the NP and CNP treatments for the 5–35 level (n = 18, p<0.044).

Phytoplankton primary production increased markedly in the NP enrichment gradient up to the 25 level, while it was lower and more stable in the CNP enrichments (Fig. 5b). The primary production was significantly higher in the NP than in the CNP treatment for the 5–35 levels (n = 18, p<0.001).

Bacterial production increased up to the 20 NP level, after which it decreased (Fig. 5c). In the CNP series the bacterial production was relatively low and more stable. A significant part of the bacterial production could be explained by variations in primary production (r^2^ = 0.39, p<0.004). Bacterial production was significantly higher in the NP than in the CNP treatments for the 5–35 levels (n = 18, p<0.012).

Pelagic food web efficiency, defined as zooplankton production divided by basal production, was relatively stable across the enrichment series (Fig. 5d). It averaged around 3% and 4% in the NP and CNP enrichments, respectively.

#### Net ecosystem production

**Figure 6:**
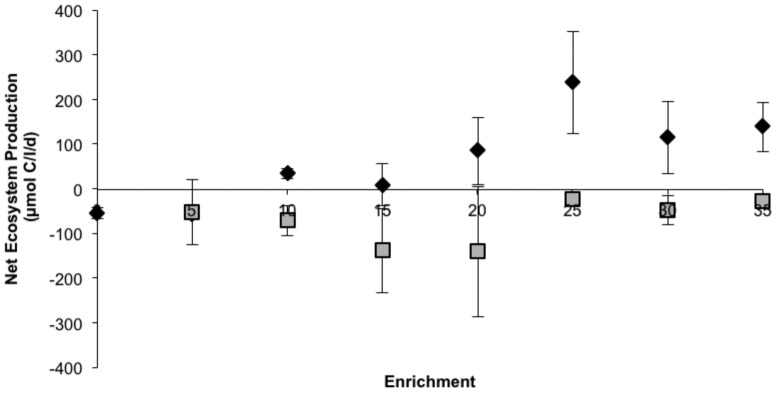
Net ecosystem production calculated during the two last weeks of the mesocosm experiment for NP (⧫) and CNP (▪) treatments. Error bars are standard error (n = 2).

During the last two weeks of the mesocosm experiment (week 4–5), respiration showed an increase both in the NP and CNP treatments to the 20 NP/CNP enrichment level, after which it levelled off or decreased (data not shown). Respiration was, however, slightly higher in the NP treatments (on average 1.2 times higher). The difference between carbon dioxide uptake (gross primary production) and respiration, i.e. the net ecosystem production (NEP), showed an increasing pattern in the NP enrichment series, while the levels were relatively low and stable in the CNP series (Fig. 6). The NP and CNP treatments differed significantly at the 5–35 level (n = 18, p<0.001).

The net ecosystem production showed positive values in the >10 NP enrichments, indicating that these systems were net-autotrophic, while in all CNP enrichments and the 0 and 5 NP treatments, negative values indicated that these systems were net heterotrophic.

### Comparison between Experimental and Natural Nutrient Concentrations

The initial nutrient concentrations in the mesocosms were lower than in the studied rivers (data not shown). However, as an effect of the daily enrichments and the relatively long water turnover time in the experimental tanks the nutrient concentrations increased in the mesocosms. In general the nutrient concentrations ended up increasing to levels more similar to those in the studied rivers, and their recipient estuaries (Table 2).

**Table 2:** Concentrations of DOC, TOC, Tot N and Tot P in enrichment water, mesocosms (final day) and natural systems (Råne, Öre and Daugava rivers) and their recipient estuaries.

Experiment	DOC* TOC**(µM)	Tot N(µM)	Tot P(µM)	C/N	C/P	N/P
**Enrichments**						
5	11.6	0.5	0.0	21	300	14
10	23.1	1.1	0.1	21	300	14
15	34.7	1.6	0.1	21	300	14
20	46.2	2.2	0.2	21	300	14
25	57.8	2.7	0.2	21	300	14
30	69.3	3.2	0.2	21	300	14
35	80.9	3.8	0.3	21	300	14
Mesocosms*						
**NP**						
0	478.3	19.4	0.4	25	1348	55
5	561.7	23.2	0.7	24	791	33
10	703.1	29.6	1.2	24	583	25
15	762.5	34.4	1.8	22	430	19
20	875.0	43.4	2.5	20	357	18
25	849.2	55.1	2.7	15	317	21
30	793.3	78.6	4.3	10	186	18
35	820.0	70.0	3.5	12	238	20
**CNP**						
5	653.3	27.9	1.0	24	675	29
10	801.4	35.0	1.2	23	665	29
15	896.7	42.6	2.0	21	448	21
20	910.0	52.2	2.4	17	386	22
25	767.3	81.4	4.2	9	183	19
30	1011.7	61.4	3.1	16	323	20
35	1038.3	95.7	4.8	11	218	20
**Natural systems**						
Råne river* a	719.4 (304)	24.6 (4)	0.3 (0.1)	29	2385	82
Råne estuary* b	571.8 (78)	27.2 (2)	0.3 (0.1)	24	2084	88
Öre river* a	806.0 (49)	26.1 (5)	0.4 (0.1)	31	2015	65
Öre estuary* b	504.6 (120)	24.3 (13)	1.0 (1.1)	21	510	24
Daugava river** c	1401.4 (20)	94.8 (32)	1.9 (0.1)	16	735	50
Gulf of Riga** d	900.0 (381)	61.7 (26)	1.3 (0.4)	15	692	48

a average of 3 measurements at 1 sampling location.

b average of 3 measurement at 6 sampling locations.

c average of 1 measurement at 6 sampling locations.

d average of 1 measurement at 5 sampling locations.

Numbers within brackets are standard deviation.

The C, N and P concentrations were higher in the southerly Daugava river than in the two northerly rivers, and the stoichiometry differed. Compared to the Daugava river, the Öre and Råne rivers contained higher carbon concentration in relation to nitrogen and phosphorus. The C:P, C:N and N:P ratios were 3, 2 and ∼1.5 times higher in the northerly than in the southerly rivers, respectively. The studied rivers had higher C to P ratios (∼700–2400) than we used in the enrichment water in the experiment (300).

## Discussion

As expected, increasing nitrogen and phosphorous load caused higher organic production in the recipient seawater, where large filamentous phytoplankton species (>20 µm) bloomed. When humic substances were added simultaneously with nitrogen and phosphorus, however, eutrophication did not occur. The traditional view that nitrogen and phosphorus load will result in the eutrophication of recipient coastal waters (e.g. [33]) may therefore be questioned in certain scenarios. Our data indicate that the organic production is regulated in a complex way, as the plankton response to nitrogen and phosphorus load is strongly influenced by a simultaneous load of carbon-rich humic substances. The results show that the C:N:P stoichiometry of the discharge water is important for the response mechanism in the recipient water.

Inferring changes in natural systems from mesocosm data is difficult due to unavoidable disruption of the food web (pumping and transfer to tanks), individual tank effects (for example 25 CNP) and experimental conditions selected for the experiment duration. Firstly, the use of HF, a commercially available humic substance, may have exposed the CNP treatments to ancillary compounds (e.g heavy metals) that were detrimental to the plankton. However, the similarity in species composition of primary producers in both the NP and CNP treatments would support a steady succession over the time course of the experiment (Fig. S3), rather than any direct toxicity in the CNP treatments. Secondly, the enrichments in our mesocosm experiment had a CNP stoichiometry of 300∶14∶1 in the CNP treatments and 0∶14∶1 in the NP enrichments (see Material and Methods and Table 2). Natural river water entering the Baltic Sea contains high concentrations of C in relation to N and P, higher than we applied in our experiment; river C:N:P stoichiometries being in the range of 735–2385∶65–82∶1. This deviation from the natural system is of relevance both to the light climate and the level of carbon available to support the heterotrophic component. On the one hand HF absorbed 16 times more PAR than the natural fulvic acids isolated from the Öre river, however, natural river water had higher C to P ratio (2–7 times) than the enrichment water used in our experiment and, in the case of the northerly rivers, higher even than the most enriched mesocosm end points (∼2 times, Table 2). We believe that a possible light inhibition effect of the relatively brown HF is at least partly balanced by the higher C to P ratio of natural river discharge, and that similar effects could be expected in natural systems influenced by NP and C (humic) influx.

Our study suggest that even if high N and P concentrations build up in the recipient water it does not lead to increased production, if the nitrogen and phosphorus load is accompanied by colored organic carbon. This inhibition effect was especially strong for algal production, which is of uttermost importance to higher trophic levels in both pelagic and benthic food webs [20], [34], [35], due to the efficiency and elevated production of phytoplankton based systems. In the CNP enrichment gradient the organic C, Tot N and Tot P increased by a factor of 2, 3 and 12, respectively, but the primary production did not increase. The phytoplankton and mesozooplankton production were ∼23 and ∼4 times higher in the NP than in the CNP enrichments, respectively.

Allochthonous carbon in the CNP enrichments may have inhibited phytoplankton primary production either by reducing the light levels in the water, thus directly hampering phytoplankton growth, or by promoting bacterial growth, thereby diverting inorganic nitrogen and phosphorus from phytoplankton to bacteria. The latter may be possible if bacteria became independent of phytoplankton carbon exudates due to the presence of an external bioavailable carbon source. In nutrient constrained environments large phytoplankton can be outcompeted for nutrients due to the larger surface to volume ratio of bacterial cells [6]. However, in this study variation in primary production could be explained more by PAR variation (r^2^ = 0.39) than nutrient levels (r^2^ = 0.00). Inorganic nutrients were replete in the CNP enrichments, but still no increase in primary production was observed. Thus, in our experiment it was mainly a decrease in PAR, which counteracted increased primary production in the CNP enrichment gradient.

It may be speculated that food web efficiency would change if the trophic state of the aquatic system shifted (*sensu* [20]). In this experimental study we spanned a large nutritional gradient, from oligotrophic to hyper-eutrophic conditions [36], but the FWE was relatively low and stable over the gradient. Previous studies have shown that bacterial based food webs, fueled by the addition of labile organic carbon, have relatively low food web efficiencies, compared to phytoplankton based food webs, where a direct link between phytoplankton and zooplankton exists [20], [37]. However, food web efficiency has been found to be influenced both by the phytoplankton size structure and the zooplankton species composition [37].

Our data are in agreement with earlier findings regarding phytoplankton communities in oligotrophic and eutrophic environments (e.g. [6]), picocyanobacteria dominated in the non-enriched control while filamentous green-algae and colony-forming cyanobacteria dominated in the eutrophic NP systems. The structure of these phytoplankton communities could partly be explained by the nutrient availability and the size-dependant uptake kinetics of different types of phytoplankton. However, a remarkable finding was that picocyanobacteria also dominated in the highly enriched CNP tanks. The explanation for this is unclear, but we speculate that they had some heterotrophic feeding mode or were well adapted to low light levels. Results by Paoli et al. [38] support these hypotheses, as they showed that picocyanobacteria of the type *Synechococcus* spp., have ∼25% heterotrophic feeding mode and that their pigment composition indicate the potential adaption to a wide range of light levels, including low light levels.

The mesozooplankton likely exerted a strong grazing pressure on the phytoplankton community, since mesozooplankton constituted the highest trophic level. The copepod community was dominated by copepodite stages of *E. affinis* (data not shown). According to Dahlgren et al. [37] their preferred food cell size range is 5–24 µm. The proportion of cells in the size range 5–20 µm was found to be lower in the NP despite a 10–fold higher primary production compared to the CNP treatments. Moreover the zooplankton net production relative to primary production was 4 and 0.8% for the NP and CNP treatment, respectively. This indicates an additional trophic level from phytoplankton to mesozooplankton in the CNP enrichments, since at each trophic level 70–90% of the carbon is lost due to excretion and respiration and sloppy feeding [11]. In the NP treatments the copepods to larger extent were feeding directly on phytoplankton compared to the CNP treatments, where they were feeding more on heterotrophic plankton as also confirmed by the low proportion of flagellates found in these treatments.

The overall low food web efficiencies (3–4%) could be explained by the dominance of <5 µm phytoplankton in the systems with no or low NP enrichment and in the humus enriched systems. In these systems the carbon had to pass via protozoa before reaching the highest trophic level, mesozooplankton. In the NP enriched systems filamentous phytoplankton became dominant, and this phytoplankton group cannot be efficiently consumed by the dominating zooplankton species *E. affinis* [37]. Thus a large proportion of the carbon flow had to pass via the microbial food web. As a result all systems had low food web efficiencies. In this experiment we did not obtain a dominance of nanoplanktonic flagellates, e.g. *Pyramimonas* spp., which in previous studies have been shown to give high food web efficiencies in the northern Baltic Sea [20], [37]. Our results support the view that the phytoplankton size spectrum plays a pivotal role for the food web efficiency in pelagic systems.

Climate change scenarios predict that precipitation will increase in the northern part of the Baltic Sea, while it may decrease in southern parts [39], [40]. However, episodic events may in the future lead to heavy periodic precipitation in southern parts. Based on our results and the stoichiometry and nutrient concentrations in the studied river water, we find it likely that climate induced increased river inflow will not cause an increase in production in the northern parts of the Baltic Sea, rather lead to net-heterotrophy in the recipient coastal system. However, the riverine CNP concentrations and stoichiometry in the southern part of the Baltic Sea indicate that increased river discharge are somewhat more likely to lead to increased phytoplankton production, eutrophication. On the other hand, based purely on this study, it is not possible to assess exactly at which CNP stoichiometry the inhibition effect occurs. Since the Daugava river contains relatively high concentrations of organic carbon, it is also possible that increased river inflow might lead to decreased pelagic production and net-heterotrophy. It is possible that the proposed “eutrophication-inhibition” mechanism associated with humic-containing river discharge might be widespread in the boreal zone of northern Europe.

Humic matter discharged into coastal systems delivers not only C but also N and P and other compounds due to its chelating properties [41]. In line with this, the addition of humic substance in our experiment caused an increase in some N and P fractions in CNP relative to NP (Table 2), indicating that the humic acid might have been a nutrient subsidy. However, the addition of humic matter was clearly shown to reduce the production at different trophic levels (Fig. 5). It is possible that long time exposure, like in aquatic systems with long water turn over times, continuous enzyme attacks and photic degradation, make the relatively refractive humic substances more bio-available which promotes heterotrophic bacterial growth [10]. In the northern Baltic Sea decreased primary production, induced by increased freshwater inflow during two years, was likely to cause a severe population crash of the benthic amphipod *Monoporeia affinis* [10], [35]. This indicates that pulses of increased freshwater inflow can have drastic and long-term ecological effects in recipient marine ecosystems.

In conclusion, this study showed the potential of humic-rich discharge water to inhibit phytoplankton growth in recipient systems. According to our results, there is no definitive coupling between nitrogen and phosphorus load and eutrophication in recipient systems. If a concomitant load of C, N and P occurs, then it may lead to decreased production of lower as well as higher trophic levels, i.e. oligotrophication. Furthermore, the species composition, size structure of the food web and food web efficiency may change, although this study does not claim to be able to predict such changes in natural systems. Although further studies are needed to clarify the detailed interactions involved, the data presented here suggest that in a climate change scenario, represented by increased precipitation and therefore increased load of C, N and P to coastal systems, it is possible that heterotrophic bacteria and the heterotrophic part of the pelagic food web would be favored, and/or the autotrophic part of the food web hindered. In such cases recipient systems would become net-heterotrophic, emitting carbon dioxide, which would exacerbate climate change scenarios.

## Supporting Information

Figure S1Changes in bacterial (a and b), phytoplankton (c and d) and zooplankton (e and f) biomass over time during the mesocosms experiments. Figures a, c and e represent NP treatments and b, d and f represent CNP treatments: 0 (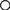), 5 (▪), 10 (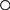), 15 (⧫), 20 (▪), 25 (×), 30 (⧫) and 35 (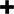).(TIF)

Figure S2 Changes in phytoplankton size class during the experiment. Figures a, b and c represent NP 5, 15 and 30, respectively, and figures d, e and f represent CNP 5, 15 and 30, respectively. Phytoplankton smaller <5 µm (black bars), 5–20 µm (dark grey bars) and >20 µm (pale grey bars).
(TIF)

Figure S3 Changes in biomass of major phytoplankton >5 µm with time: a) week 1, b) week 3 and c) week 5.
(TIF)
