# Supplementary figures and images for: Can Humic Water Discharge Counteract Eutrophication in Coastal Waters?
Source: PLoS One. 2013 Apr 18;8(4):e61293. doi: 10.1371/journal.pone.0061293 (PMC3630215; doi:10.1371/journal.pone.0061293)

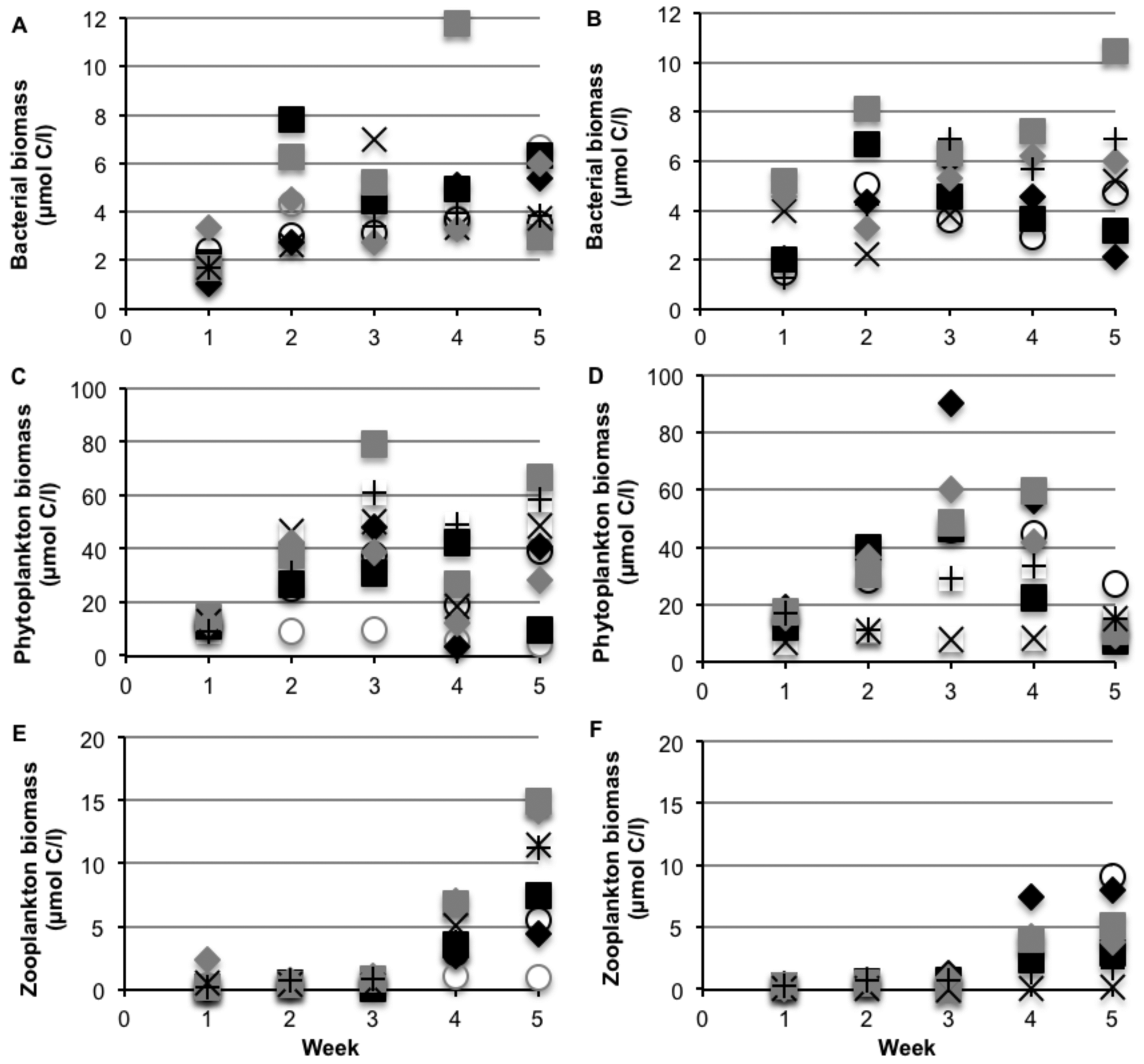

Supplement: Figure S1 — Changes in bacterial (a and b), phytoplankton (c and d) and zooplankton (e and f) biomass over time during the mesocosms experiments. Figures a, c and e represent NP treatments and b, d and f represent CNP treatments: 0 (), 5 (▪), 10 (), 15 (⧫), 20 (▪), 25 (×), 30 (⧫) and 35 (). (TIF) [file pone.0061293.s001.tif]

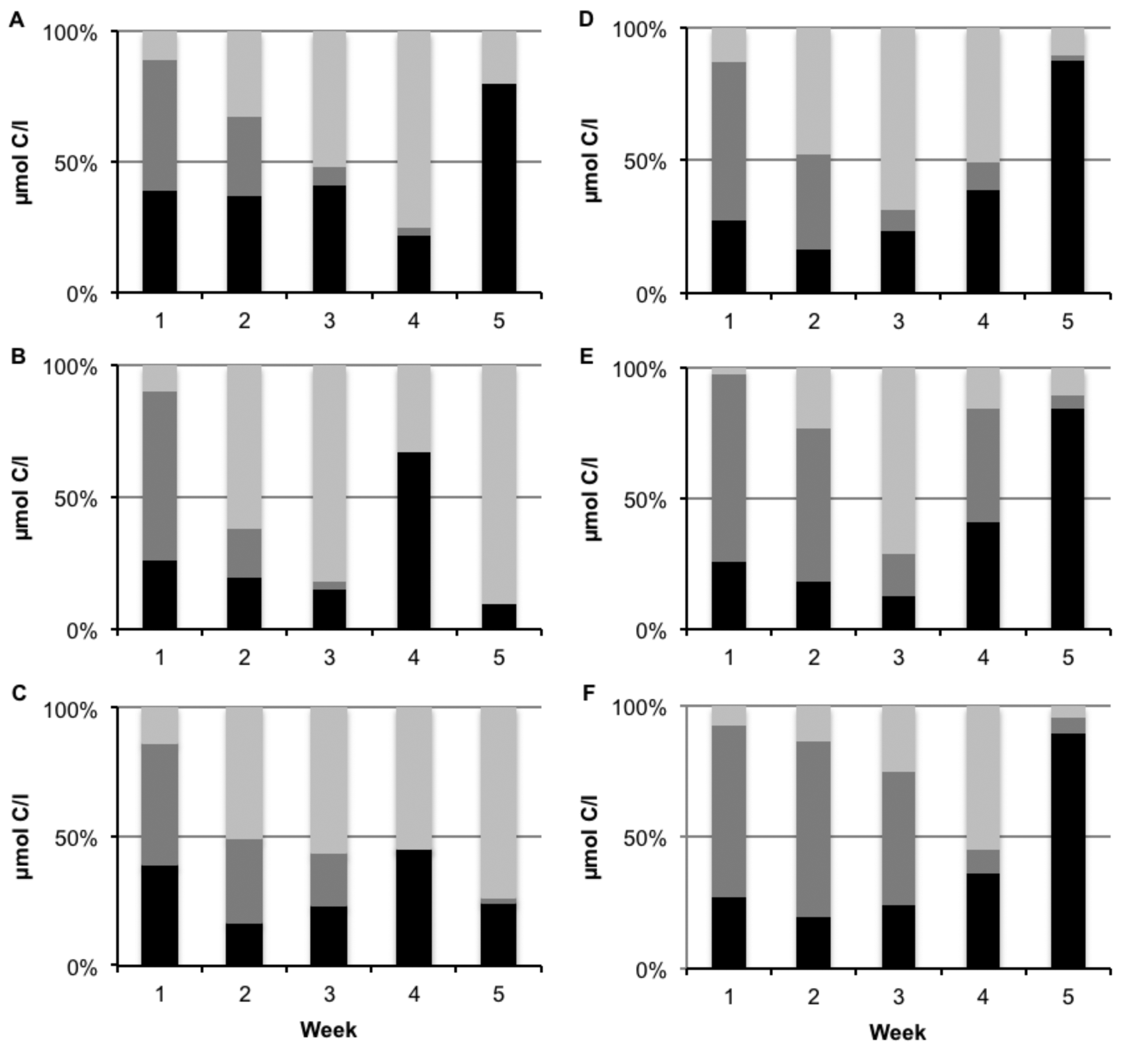

Supplement: Figure S2 — Changes in phytoplankton size class during the experiment. Figures a, b and c represent NP 5, 15 and 30, respectively, and figures d, e and f represent CNP 5, 15 and 30, respectively. Phytoplankton smaller <5 µm (black bars), 5–20 µm (dark grey bars) and >20 µm (pale grey bars). (TIF) [file pone.0061293.s002.tif]

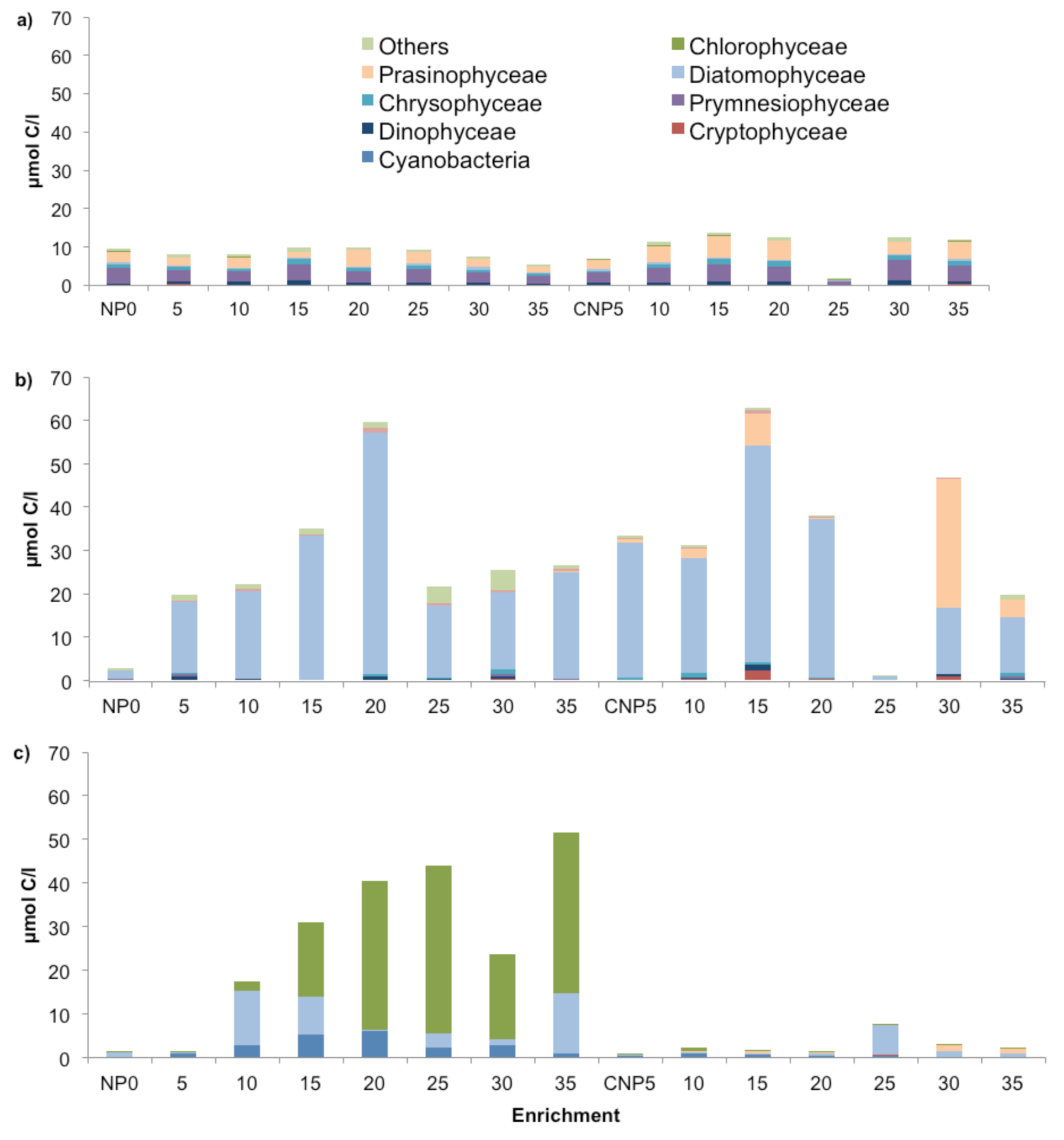

Supplement: Figure S3 — Changes in biomass of major phytoplankton >5 µm with time: a) week 1, b) week 3 and c) week 5. (TIF) [file pone.0061293.s003.tif]
